# SERS active silver nanoparticles synthesized by inkjet printing on mesoporous silicon

**DOI:** 10.1186/1556-276X-9-527

**Published:** 2014-09-25

**Authors:** Chiara Novara, Francesco Petracca, Alessandro Virga, Paola Rivolo, Sergio Ferrero, Alessandro Chiolerio, Francesco Geobaldo, Samuele Porro, Fabrizio Giorgis

**Affiliations:** 1 Department of Applied Science and Technology, Politecnico di Torino, C.so Duca degli Abruzzi 24, 10129 Torino, Italy; 2 Istituto Italiano di Tecnologia, Center for Space Human Robotics, C.so Trento 21, 10129 Torino, Italy

**Keywords:** Inkjet printing, Surface-enhanced Raman scattering, Plasmonic nanoparticles, Porous silicon, Organic dyes

## Abstract

Inkjet printing technique is exploited for the synthesis of Ag nanoparticles (NPs) patterned on electrochemically etched silicon-based substrates. The nanostructure morphology, here analyzed by scanning electron microscopy, is dictated by the ink composition and the printing parameters. Under suitable excitation conditions, resonant surface-enhanced Raman scattering (SERS) performed on such metal-dielectric nanostructures can approach single-molecule detection as recently demonstrated on silvered porous silicon synthesized by immersion plating.

**PACS:**

78.67.Bf; 78.30.-j

## Background

Surface-enhanced Raman scattering is a powerful sensing technique, whose great efficiency is due to the amplification of the Raman signal yielded by molecules either adsorbed on or close to noble metal nanoparticles [1]. Actually, the increase of the Raman scattering efficiency can be attributed to the following processes: (i) an enhancement of the electromagnetic (EM) fields localized at the edges of metallic particles (Raman hot spots) after excitation of localized surface plasmons (LSPs) at resonance conditions, (ii) a charge-transfer (CT) mechanism wherein an electron can be transferred from an excited metal state to a vibrational level within the target molecule. The EM enhancement is much more efficient than the CT mechanism, but sometimes their coexistence can even lead to single-molecule detection [2,3].

While solving the limitations of the low Raman scattering cross section of most molecules, surface-enhanced Raman scattering (SERS) keeps the advantages of Raman spectroscopy, providing a vibrational fingerprint of the analyzed species and being compatible with biotechnology applications in water-based environments [4].

In the framework of multiplexing sensors and biosensors development, inkjet printing represents a low-cost, additive patterning technique suitable for the deposition of metal nanoparticles assembled in functional arrayed elements constituting efficient SERS substrates. By means of a servo-assisted piezoelectric printhead, fed with an engineered ink, microdroplets can be ejected on flexible/rigid substrates drawing complex structures with controlled geometry characterized by quite high spatial resolution [5].

In a previous work, the *in situ* synthesis of silver nanoparticles by inkjet printing on mesoporous silicon was demonstrated [6]. A silver nitrate-based ink was printed on the Si-H-covered porous silicon surface where Ag^+^ cations were reduced to form silver nanoparticles, exploiting the same reaction involved in the consolidated immersion plating technique where porous silicon substrates are dipped in different noble metal ion solutions [7].

In this work, the inkjet printing synthesis of Ag nanoparticles (NPs) is optimized aiming at the development of efficient SERS substrates characterized by a high Raman enhancement, satisfying uniformity and good reproducibility. The main process parameters are discussed in terms of their influence on the abovementioned features.

## Methods

### Porous silicon synthesis and patterning of Ag nanoparticles

Highly boron-doped silicon wafers with a resistivity of 34 mΩ-cm were used as starting material. Room temperature anodization was performed in HF solution (20:20:60 HF/H_2_O/CH_3_CH_2_OH) with a current density of 125 mA/cm^2^ for 30 s, producing homogeneous single layers of mesoporous silicon. The thickness of the film was 1.7 μm with 64% of porosity. Two different solvent mixtures (water/ethanol (WE) 1:1 and water/dimethyl sulphoxide (DMSO) 3:1 + ethanol (WDE) 10% vol) were used to prepare inks at variable AgNO_3_ concentrations (2.5 10^-2^ M, 5 10^-2^ M).

The inks were printed at room temperature (for WE) and at 65°C by heating the substrate plate of the inkjet printer (for WDE), using a piezoelectric Jetlab 4-XL printer from MicroFab Technologies Inc. (Plano, TX, USA) equipped with a 60-μm nozzle diameter MJ-AT-01 dispenser. For the WE ink, the waveform used to eject a single droplet was a 35-V pulse lasting 24 μs, followed by a -35-V pulse lasting 80 μs as echo dwell with the rise, fall, and final rise time of 13, 23, and 10 μs, respectively; for the WDE mixture, the waveform consisted in a 26-V pulse lasting 20 μs, followed by a -35-V pulse lasting 40 μs as echo dwell with the rise, fall, and final rise time of 21, 13, and 5 μs, respectively. The step size (drop-to-drop distance) and the number of printhead passes were parameterized.

### SERS substrate characterization

Scanning electron microscopy images of silvered porous silicon samples were obtained as secondary electron contrast images with 5-keV electrons using an in-lens detector of a Zeiss SUPRA 40 (Zeiss SMT, Oberkochen, Germany) field emission electron microscope (FESEM).

Specular reflection spectra were acquired using an Agilent Cary 5000 (Agilent, Santa Clara, CA, USA) UV-visible-NIR spectrophotometer equipped with a 12.5° reflectance unit, in the 200-to-1,500-nm range.

Raman spectra were obtained by means of a Renishaw micro-Raman spectrophotometer equipped with a charge-coupled device camera. Samples were excited with an Ar-Kr laser source (514.5 nm), providing a photon flux lower than 60 W/cm^2^ by a × 50 microscope objective (numerical aperture 0.75, light spot diameter <2 μm). The spectral resolution and integration time were 3 cm^-1^ and 10 s, respectively.

Organic dyes (Cyanine Cy5 and Rhodamine R6G) dissolved in ethanol solutions at several molar concentrations were used to check the SERS response of the synthesized metal-dielectric nanostructures. The samples were dipped into the dye solution for 30 s and then left to dry before the Raman measurement. The presented Raman spectra were baseline-corrected by removing the background fluorescence of the analyzed dyes.

## Results and discussion

**Figure 1:**
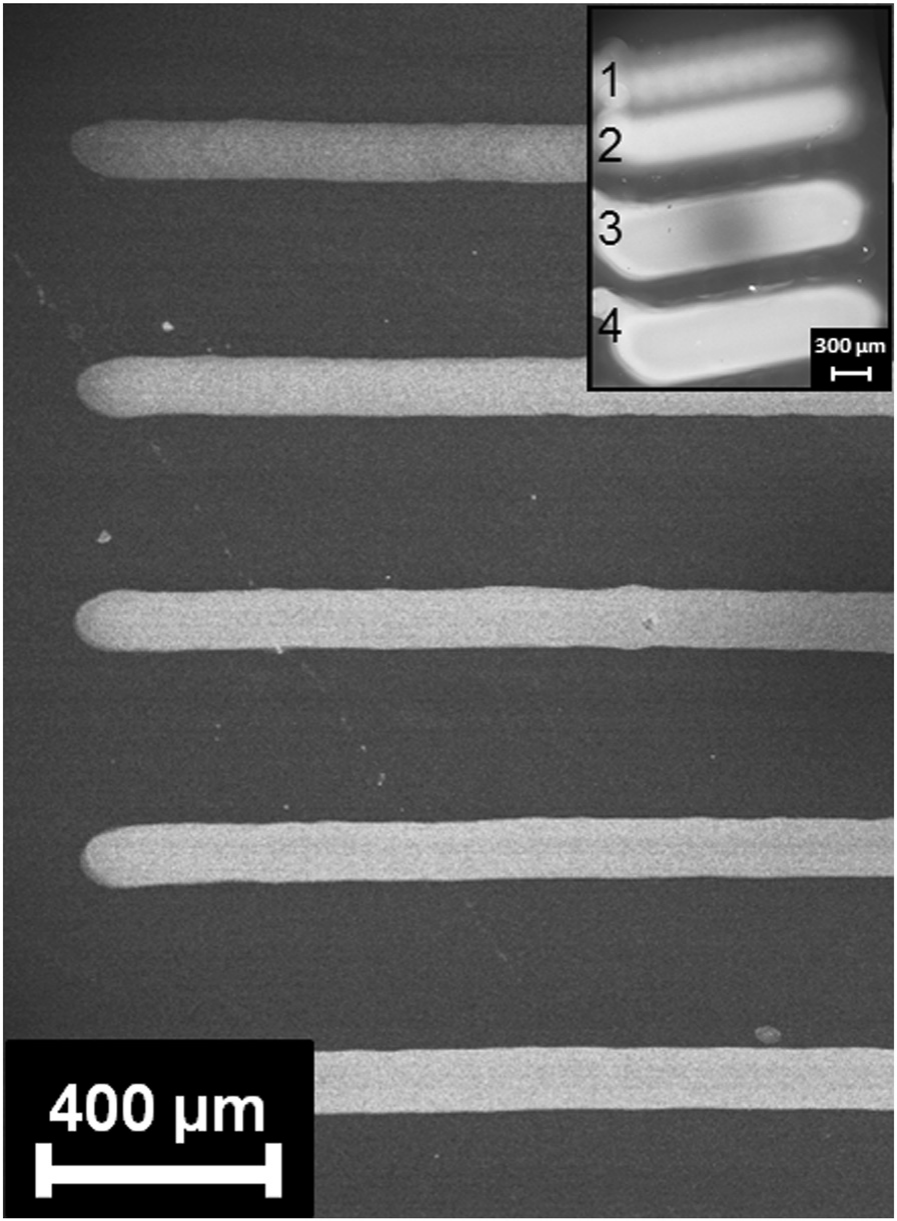
**Ag nanoparticles pattern on porous silicon.** FESEM image of a patterned sample. The inset shows the effect of the inkjet step size on the uniformity of the stripes: (1) 130 dpi, (2) 260 dpi, (3) 1,000 dpi and (4) 1,300 dpi.

Unlike most of the inkjet printing applications, where NPs suspended in the ink are directly deposited onto the substrate, this work concerns with their synthesis on porous silicon through the ‘in situ’ reduction of silver cations by printing AgNO_3_ solutions on the reactive hydrides covered porous silicon surface [8].For all the discussed experiments, the inkjet printer was set in the drop-on-position (DOP) modality, which enables the control over the ink amount dispensed for each single spot. A typical stripe pattern of silver NPs on porous silicon is shown in Figure 1. Lines are formed by adjacent droplet coalescence and their width is mainly determined by the solvent content of the ink and by the droplet contact angle with the porous silicon surface. Typical lateral width is 400 μm for the water-ethanol ink and 80 to 100 μm for the DMSO-containing one, with oscillations caused by the different number of passes and in particular by the step size, that also influences the uniformity of the line, as shown in the inset.

Raman enhancement yielding SERS effect is strictly dependent on the morphology and spatial arrangement of the metal NPs [9]. In our case, the control over such features can be achieved by the adjustment of the printing parameters as well as by the suitable choice of the ink composition.

**Figure 2:**
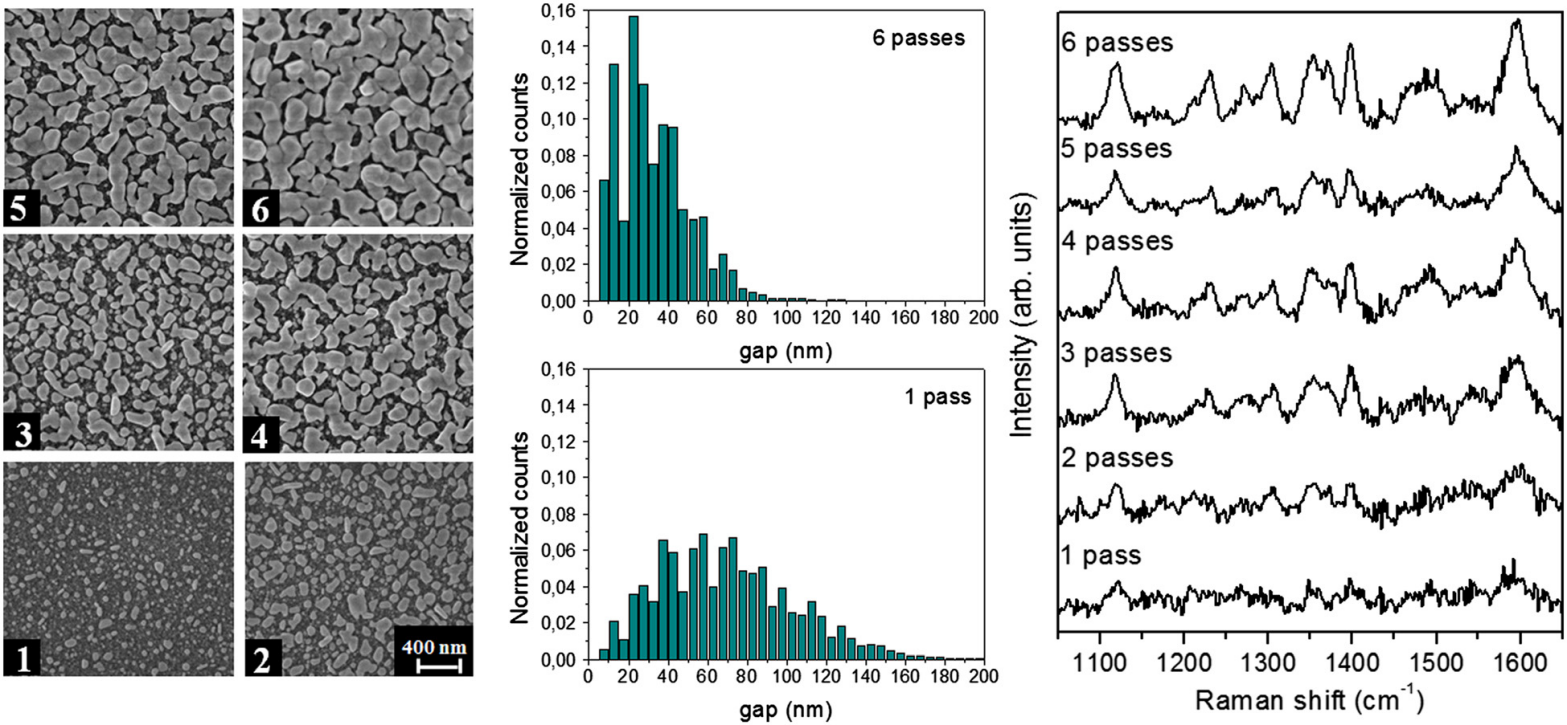
**Effect of the number of passes: AgNO**_**3 **_**2.5 10**^**-2 **^**M.** Left: FESEM images of Ag nanoparticles printed with WE ink, 1,000 dpi, variable number of passes of the printhead on the silicon substrate, 1 to 6. Center: inter-particle gap distribution for 1 and 6 passes. Histograms are normalized with respect to the total number of counts. Right: SERS spectra of Cy5 (10^-6^ M) adsorbed on the same six lines.

**Figure 3:**
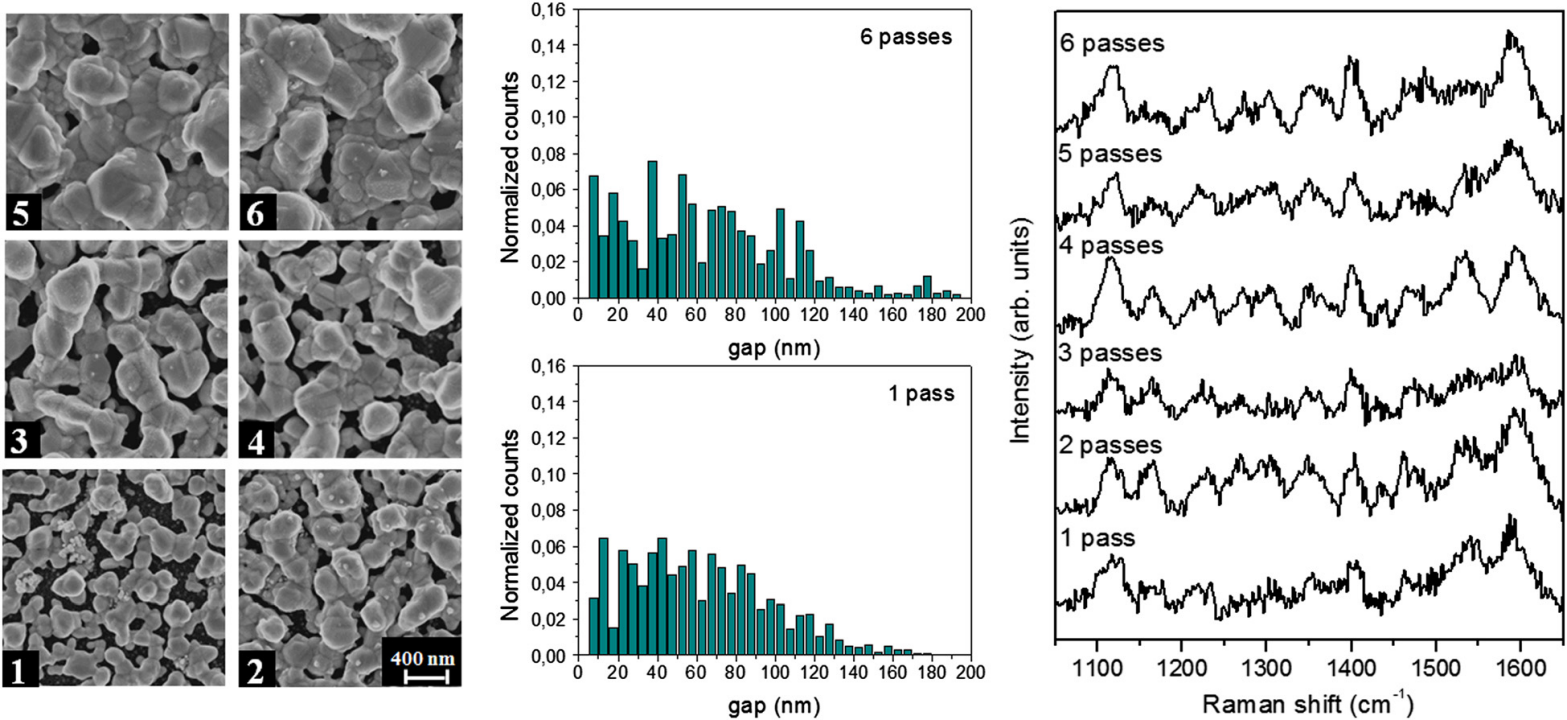
**Effect of the number of passes: AgNO**_**3 **_**5 10**^**-2 **^**M.** Left: FESEM images of Ag nanoparticles printed with WE ink, 1,000 dpi, variable number of passes of the printhead on the silicon substrate, 1 to 6. Center: inter-particle gap distribution for 1 and 6 passes. Histograms are normalized with respect to the total number of counts. Right: SERS spectra of Cy5 (10^-6^ M) adsorbed on the same six lines.

Figures 2 and 3 illustrate the correlation between the NP morphology and their SERS response, when the number of passes of the printhead on the silicon substrate is increased. A MATLAB routine for FESEM images analysis has been developed in order to get an insight on the nanostructure morphological evolution by changing the inkjet printing conditions. FESEM viewgraphs at × 100,000 magnification are used as input for the image analysis of each line. The distributions of the gap between neighboring NPs are obtained by applying the distance transform function to the FESEM images, a MATLAB operator that replaces each background pixel value with the distance from the closest NP, considering the local maxima of the obtained map as the half gap value at each point. Among the output parameters computed by the MATLAB script, we analyzed the coverage percentage, namely the fraction of Ag coated surface, and the average inter-particle gap. This choice is driven by the fundamental role played by the inter-particle distance in a SERS substrate. Actually, the greatest SERS enhancement occurs at hot spots, where the coupling of LSPRs within two (or more) very close NPs yields a huge concentration of the EM field [10]. In particular, at resonance condition, the highest Raman enhancement is obtainable for the smallest inter-particle gap size [11].

Table 1 summarizes the results of the image analysis for samples shown in Figures 1 and 2, concerning with nanostructures obtained with different AgNO_3_ concentration in the ink. For each added ink pass, the Ag coverage as well as the size of the NPs increases. Indeed, at lower AgNO_3_ concentration (2.5 10^-2^ M), the inter-particle gap distribution is quite wide for a single printhead pass and it becomes narrower and narrower as the number of passes increases; at the same time, the average gap decreases and the particles size increases. On the other side, for higher AgNO_3_ concentration (5 10^-2^ M), despite the increase of the average particle size and of the Ag coverage, the gap distribution between the particle protrusions and its average value are almost unaffected by the ink amount reacting with the porous silicon substrate (i.e., the number of printhead passes). Such a behavior suggests a growth mechanism, in which the forming smaller gaps are rapidly filled, with the coalescence of the bigger NPs.

**Table 1:** FESEM images numerical analysis

**Number of passes**	**1**	**2**	**3**	**4**	**5**	**6**
Concentration 2.5 10^-2^ M						
Coverage%	13	48	58	72	75	80
Average inter-particle gap (nm)	89	43	39	36	34	28
Concentration 5 10^-2^ M						
Coverage%	70	91	89	89	96	97
Average inter-particle gap (nm)	60	44	65	68	64	62

Output parameters for lines printed with WE ink, 1,000 dpi, and variable number of passes, for two different silver nitrate concentrations.

The features of the inter-particle gap size distribution are clearly reflected in the SERS efficiency of the metal-dielectric nanostructures. In order to check such efficiency, the structures shown in the FESEM viewgraphs of Figures 2 and 3 were impregnated with Cyanine Cy5 dissolved in ethanol solutions at a concentration of 10^-6^ M. The Raman spectra are characterized by the typical Cy5 bands at 1,605, 1,500, 1,230, and 1,120 cm^-1^ corresponding to *v*(C = N)_stretch_, *v*(C = C)_ring-stretch_, *v*(C-N)_stretch_, and *v*(C-H)_ip-bend_ modes of the dye, respectively. All the nanostructures provide a SERS effect, as the spectrum of Cy5 cannot be detected on non-silvered porous silicon until a 10^-2^ M Cy5 solution is employed for the impregnation. It is straightforward to notice as for the inks with the lower AgNO_3_ concentration, the Raman signal of the organic dye shows a monotonic intensity increase by increasing the number of printhead passes, which can be attributed to the efficient Raman hot spots occurring within the smallest inter-particle gaps. For the ink with the higher AgNO_3_ concentration, this effect is much less evident because of the similar gap distribution verified for all the samples obtained with different number of passes.

**Figure 4:**
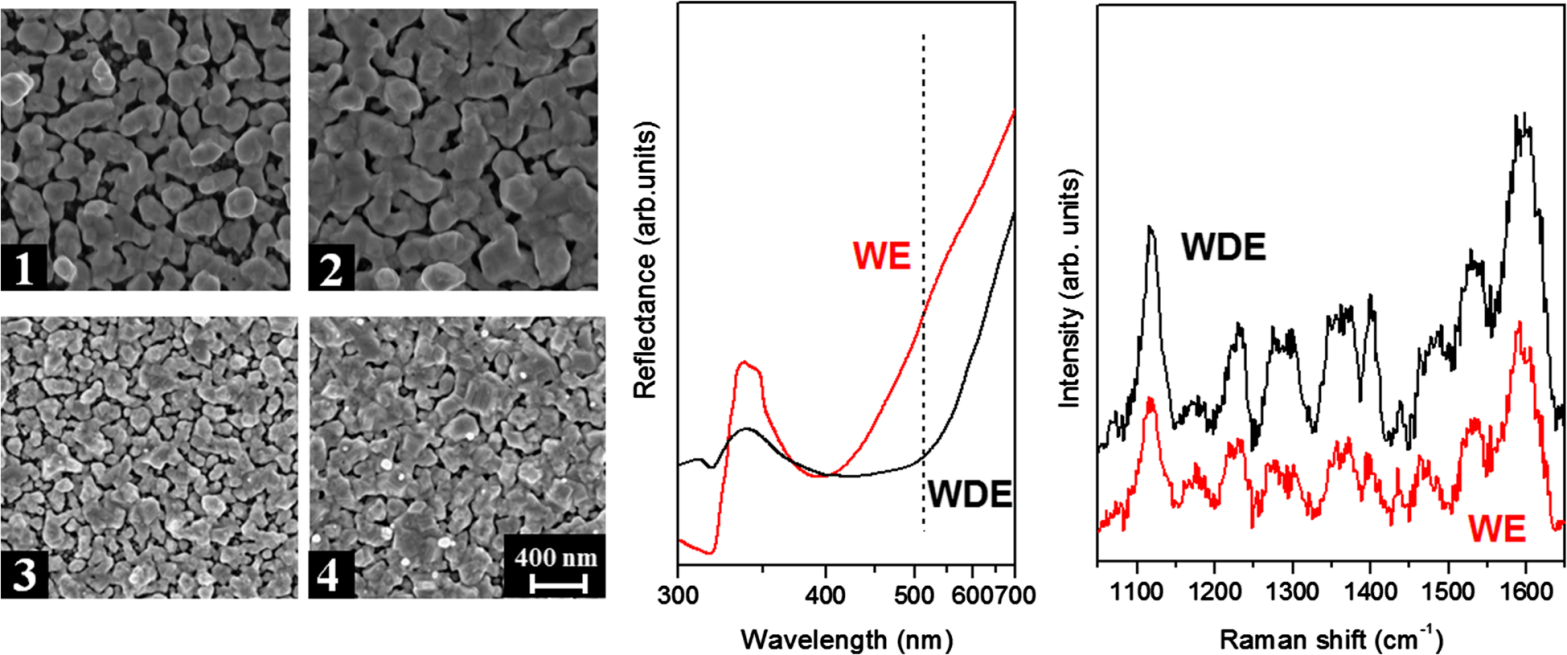
**Solvent effect on NPs morphology.** Left: FESEM images of silver nanoparticles obtained printing AgNO_3_ 2.5 10^-2^ M (1 to 2) water-ethanol ink, 4 and 6 passes (3 to 4) water/DMSO/ethanol ink, 4 and 6 passes. Center: UV-vis specular reflectance spectra showing the LSPRs dips of the lines printed with the WE and WDE inks (6 passes). The dashed line indicates the excitation laser wavelength at 514.5 nm used in the Raman experiment. Right: SERS spectra of Cy5 (10^-6^ M) adsorbed on the same lines.

A complex morphology variation is observed changing the ink composition, moving from the WE to the WDE ink. It is worth to highlight that the addition of a quite low amount of DMSO yields a variation of the chemical-physical properties of the AgNO_3_ solution such as the viscosity and the surface tension, thus improving the spatial resolution of the designed pattern and providing an enhanced ink printability which prevents satellite drops ejection. In Figure 4, FESEM images of the silver NPs synthesized with the two inks typologies are compared (corresponding image analysis is presented in Table 2). For the same nominal inkjet printing parameters, larger and surface-smoothed NPs are synthesized using the WE ink, whereas sharper edges and smaller sizes are obtained printing the WDE mixture.UV-vis specular reflectance spectra (Figure 4, center) are recorded in order to analyze the influence of the different morphologies on the optical response in terms of LSP resonances. The spectra show marked dips related to plasmonic inter-particle short-range interactions due to dimer NP assemblies. While the spectrum for the WE sample shows a quite narrow dip centered at about 400 nm, the silver NPs synthesized with the WDE ink are characterized by a very broad resonance. Interestingly, using a laser excitation wavelength of 514.5 nm, which is far from the dye absorption band (off-electronic resonance condition), the SERS spectra obtained for a spotted Cy5 solution with the same molar concentration yield a greater enhancement for the WDE sample: the signal intensity is almost twice that for the WE one. This difference can be reasonably explained since the laser excitation falls within the plasmonic band of the WDE-synthesized substrate, while in the WE one, the excitation concerns with the reflectance dip tail (plasmonic resonance condition not properly fulfilled for WE substrate).

**Table 2:** Comparison between the WE and WDE ink

**Number of passes**	**4**	**6**
Water/ethanol		
Coverage%	81	89
Average inter-particle gap (nm)	36	28
Water/DMSO/ethanol		
Coverage%	85	93
Average inter-particle gap (nm)	22	21

Output parameters of the gap analysis for samples shown in Figure 4.

As the WDE sample exhibits the better performances under our excitation conditions, it is used to evaluate the lowest Raman limit of detection achievable on inkjet-printed plasmonic stripes. With this aim, Rhodamine 6G is chosen as a probe molecule because it offers the possibility to work in surface-enhanced resonant Raman scattering (SERRS) regime. Actually, the HOMO-LUMO electronic transition of the dye occurs around 520 nm, matching the used excitation wavelength and being in this mode in resonance both with the plasmonic substrate and with the analyte electronic transitions.

**Figure 5:**
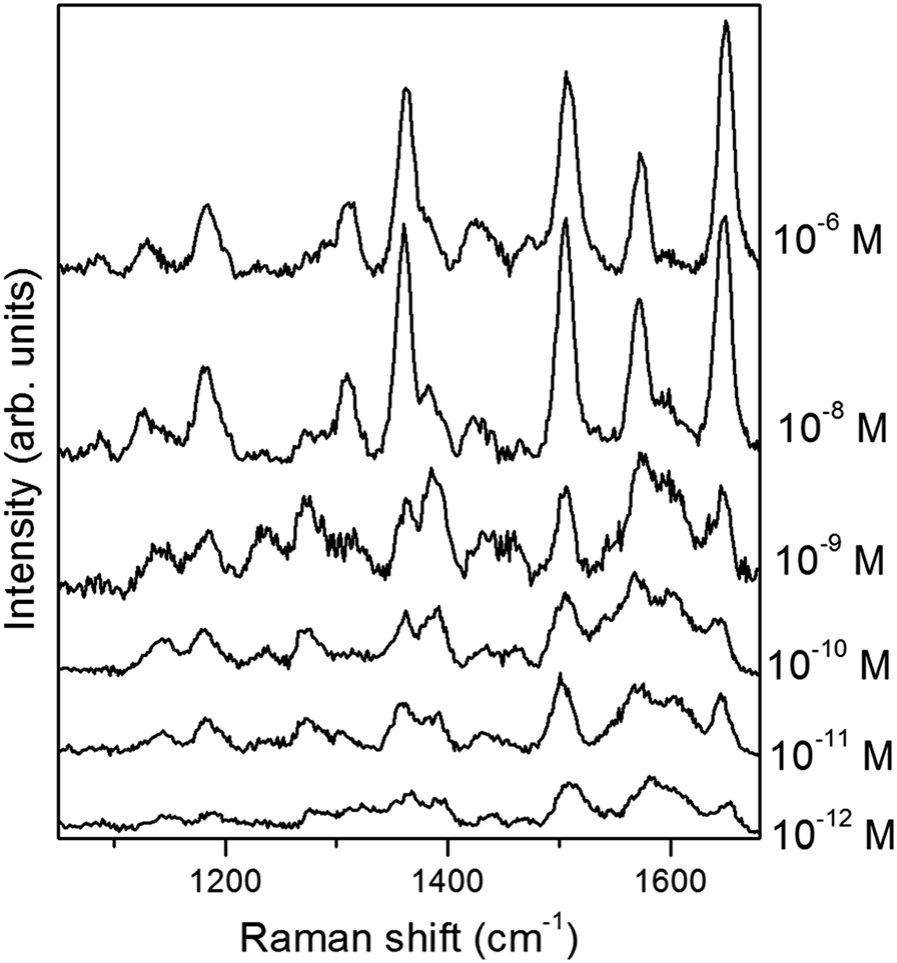
**Limit of detection evaluation.** SERRS spectra of Rhodamine 6G adsorbed from solutions at different dye concentrations (10^-12^ to 10^-6^ M) on a line printed with WDE ink, AgNO_3_ 2.5 10^-2^ M, 1,000 dpi, six passes.

SERS spectra collected after dipping the substrates in solutions at increasing dye concentrations are plotted in Figure 5. The 1,647- and 1,509-cm^-1^ modes, assigned to the C-C stretching of the xanthene ring [12], are easily detected until 10^-12^ M concentration. Moreover, also at the lower concentrations, the whole vibrational pattern of Rhodamine 6G is still present, although some changes in the intensity ratios are observed for bands in the 1,200-to-1,400-cm^-1^ range.

Following a previous study, we can evaluate an external amplified Raman efficiency (EARE), defined as the ratio between the dye concentration threshold, which is detectable on the Ag NPs, with respect to the minimum concentration of the detected analyte adsorbed on a bare p-Si substrate [13]. In our case, since the Raman spectrum of rhodamine cannot be detected after immersion of bare porous silicon in dye solutions with concentration as high as 10^-4^ M, an EARE larger than 10^8^ can be foreseen for this optimized SERS substrate. It is worth to underline that such performances are comparable to those of immersion plated silvered porous silicon which approached single-molecule detection in SERRS regime [14]. As a matter of fact, considering a micrometric laser spot size as the used optical probe and neglecting eventual dye accumulation effects in pores or nanometric gaps of the substrate, single-molecule detection can be expected for concentration lower than 10^-11^ M, as in our case.

Beside high Raman enhancements, a SERS substrate needs to satisfy other severe requirements in terms of nanostructure uniformity and reproducibility [15]. In the framework of inkjet-printed plasmonic patterns, the homogeneity of the samples has been evaluated recording several SERS spectra of Cy5 diluted at a concentration of 10^-5^ M along different sites along the same line, while the reproducibility has been analyzed comparing the intensity of the Raman spectra acquired on different lines synthesized under the same nominal inkjet printing conditions. Using the same excitation conditions, the integrated intensity of the 1,120-cm^-1^ peak corresponding to the *v*(C-H)_ip-bend_ mode of Cy5, calculated from the measurements performed on five probed spots (with a scanned length of 2 mm) on lines prepared with the WDE ink (AgNO_3_ concentration of 2.5 10^-2^ M, six passes, and 1,000 dpi), shows fluctuation lower than 20%. The intensity variation of the same Raman band acquired on four different lines (considering for each stripe the average intensity obtained for the previously discussed five spots) is lower than 15%.

## Conclusions

Arrayed SERS stripes are realized by inkjet printing on mesoporous silicon. By optimizing the nanostructure morphology in terms of densely packed silver NPs, huge Raman enhancements (EARE > 10^8^) are obtained exploiting hot spots located in between the particles.

The printed plasmonic patterns show a good uniformity and reproducibility, making the discussed fabrication technique suitable to develop active sensing platforms aimed to multiplexed label free biodetection.

## Abbreviations

NPs: nanoparticles; SERS: surface-enhanced Raman scattering; EM: electromagnetic; LSP: localized surface plasmon; CT: charge transfer; DMSO: dimethyl sulphoxide; WE: water-ethanol; WDE: water-DMSO-ethanol; DOP: drop on position; FESEM: field emission scanning electron microscopy.

## Competing interests

The authors declare that they have no competing interests.

## Authors' contributions

CN performed most of the inkjet synthesis and the Raman measurements and edited the paper. FP performed the numerical analysis of FESEM images and some of the inkjet synthesis. AV performed Raman and FESEM measurements and numerical analysis. SF performed some Raman measurements. SP performed some of the inkjet synthesis. AC organized the experiments with the inkjet printer. PR and FrG designed the experiment. FaG also designed the experiment, executed the data analysis, and edited the paper. All authors read and approved the final manuscript.

## Authors' information

CN received her master’s degree in Advanced Chemical Methodologies at the University of Torino in 2012. She is now a PhD student in Materials Science and Technology at Politecnico di Torino, where she is working on metal-dielectric substrates for surface-enhanced Raman scattering (SERS) applications, with a special focus on inkjet printing as synthesis technique.

FP received his degree in Biomedical Engineering at Politecnico di Torino in 2013. In the same year, he joined the Laboratory of Engineering of Neuromuscular System and Motor Rehabilitation (LISiN) research group, in the field of electromyographic (EMG) signal analysis and applied research. He is also involved in the study and development of mathematical models for the interpretation of simulated EMG signals.

AV received his degree in Chemical Engineering at Politecnico di Torino in 2005 and his Ph.D. in Physics in 2011. The main field of interest is the production of silver and gold nanostructures, prepared through either nanosphere lithography or deposition, for SERS substrates.

PR is an assistant researcher at the Applied Science and Technology Department of Politecnico di Torino (Italy), where she operates in the Materials and Microsystems Laboratory, a laboratory of Politecnico di Torino. In 1999, she obtained her master’s degree in Chemistry. In 2003, she took her Ph.D. degree in Materials Science and Technology. Her research activities are focused on surface chemical modification of several materials (also porous and nanostructured), even by means of plasma-assisted techniques, in order to obtain hybrid organic-inorganic materials to be applied as optical and electromechanical sensors mainly for biological diagnostics.

SF is an assistant professor at the Applied Science and Technology Department of Politecnico di Torino. He obtained his master’s degree in Electronic Engineering in 1996 and the Ph.D. in Electronic Devices in 2002. His current area of research includes optical spectroscopy and electrical characterization of semiconductors. He is the author and co-author of more than 50 scientific papers published on peer-reviewed international journals.

AC received his degree in Materials Engineering at Politecnico di Torino in 2005 and his Ph.D. in Electron Devices in 2009. He joined the χ-Lab Materials and Microsystems Laboratory research group, developing spintronic devices, MEMS, and nanocomposite materials for electronic applications. AC is currently coordinating the technologies in support of the Smart Materials platform at Istituto Italiano di Tecnologia, with the aim of realizing a smart sensing skin. He is the co-author of more than 50 scientific papers, 4 national patents, 2 international patents, and 13 book chapters and editor of 1 book.

FrG is a full professor at the Applied Science and Technology Department of Politecnico di Torino. He obtained his master’s degree in Industrial Chemistry in 1990 and his Ph.D. in Chemical Science in 1995. His current area of research includes the synthesis and characterization of materials with special attention devoted to porous silicon for several applications. He is the author and co-author of more than 110 scientific papers published on peer-reviewed international journals.

SP is a researcher at Istituto Italiano di Tecnologia focusing on CVD of nanostructured materials, inkjet printing technology, and development of advanced materials and devices. He received his MSc in Materials Science in 2001 and PhD in Physics in 2005, with a thesis on fabrication and characterization of silicon carbide power electronic devices. During and after his PhD, he has worked at the University of Linkoping (Sweden), Politecnico di Torino (Italy), and Heriot-Watt University of Edinburgh, Scotland (UK), working respectively on silicon carbide electronic devices, CVD synthesis and application of carbon nanotubes, CVD synthesis of ultrananocrystalline diamond layers and application as plasma-facing materials for nuclear fusion, nanomaterials for up-conversion of sub-band-gap photons to increase solar cell efficiencies. He has co-authored over 45 articles in international peer-reviewed journals.

FaG is an associate professor of Physics of Condensed Matter at the Applied Science and Technology Department of Politecnico di Torino where he is in charge as research delegate. He obtained his master’s degree in Physics in 1991 and his Ph.D. in Solid State Physics in 1995. He is a senior researcher at the χ-Lab Materials and Microsystems Laboratory of Politecnico di Torino and at the Istituto Italiano di Tecnologia - Center for Space Human Robotics. His current area of research concerns with the synthesis and characterization of silicon-based thin films in amorphous, microcrystalline and porous phase with applications in photonic and plasmonic nanostructures. He is the author and co-author of more than 140 scientific papers published on peer-reviewed international journals.

## References

[B1] Stiles PL Dieringer JA Shah NC Van Duyne RP Surface-enhanced Raman spectroscopy Annu Rev Anal Chem 2008 1 601 626 10.1146/annurev.anchem.1.031207.112814 20636091

[B2] Nie SM Emory SR Probing single molecules and single nanoparticles by surface-enhanced Raman scattering Science 1997 275 1102 1106 10.1126/science.275.5303.1102 9027306

[B3] Kneipp K Wang Y Kneipp H Perelman LT Itzkan I Dasari RR Feld MS Single molecule detection using surface-enhanced Raman scattering (SERS) Phys Rev Lett 1997 78 9 1667 1670 10.1103/PhysRevLett.78.1667

[B4] Kneipp K Kneipp H Itzkan I Dasari RR Feld MS Surface-enhanced Raman scattering and biophysics J Phys Condens Matter 2002 14 R597 R624 10.1088/0953-8984/14/18/202

[B5] Tekin E Smith PJ Schubert US Inkjet printing as a deposition and patterning tool for polymers and inorganic particles Soft Matter 2008 4 703 713 10.1039/b711984d 32907172

[B6] Chiolerio A Virga A Pandolfi P Martino P Rivolo P Geobaldo F Giorgis F Direct patterning of silver particles on porous silicon by inkjet printing of a silver salt via in-situ reduction Nanoscale Research Letters 2012 7 502 10.1186/1556-276X-7-502 22953722 PMC3526385

[B7] Panarin AY Terekhov SN Kholostov KI Bondarenko VP SERS-active substrates based on n-type porous silicon Appl Surf Sci 2010 256 6969 6976 10.1016/j.apsusc.2010.05.008

[B8] Tsuboi T Sakka T Ogata YH Metal deposition into a porous silicon layer by immersion plating: influence of halogen ions J Appl Phys 1998 83 4501 4506 10.1063/1.367212

[B9] Canamares MV Garcia-Ramos JV Sanchez-Cortes S Castillejo M Oujja M Comparative SERS effectiveness of silver nanoparticles prepared by different methods: A study of the enhancement factor and the interfacial properties J of Colloid and Interf Sci 2008 326 103 109 10.1016/j.jcis.2008.06.052 18656205

[B10] Krenn JR Weeber JC Dereux A Bourillot E Goudonnet JP Direct observation of localized surface plasmon coupling Phys Rev B 1999 60 5029 5033 10.1103/PhysRevB.60.5029

[B11] Le Ru EC Etchegoin PG Phenomenological local field enhancement factor distributions around electromagnetic hot spots J Chem Phys 2009 130 181101 181104 10.1063/1.3138784 19449901

[B12] Vösgrone T Meixner AJ Surface- and resonance-enhanced micro-Raman spectroscopy of xanthene dyes: from the ensemble to single molecules Chem Phys Chem 2005 6 154 163 15688659 10.1002/cphc.200400395

[B13] Virga A Rivolo P Descrovi E Chiolerio A Digregorio G Frascella F Soster M Bussolino F Marchiò S Geobaldo F Giorgis F SERS active Ag nanoparticles in mesoporous silicon: detection of organic molecules and peptide-antibody assays J Raman Spectros 2012 43 730 736 10.1002/jrs.3086

[B14] Virga A Rivolo P Frascella F Angelini A Descrovi E Geobaldo F Giorgis F Silver nanoparticles on porous silicon: approaching single molecule detection in resonant SERS regime J Phys Chem C 2013 117 20139 20145 10.1021/jp405117p

[B15] Bandarenka H Artsemyeva K Redko S Panarin A Terekhov S Bondarenko V Effect of swirl-like resistivity striations in n^+^-type Sb doped Si wafers on the properties of Ag/porous silicon SERS substrates Phys Status Solidi C 2013 10 624 627 10.1002/pssc.201200731

